# Effect of human amniotic epithelial cells on pro‐fibrogenic resident hepatic cells in a rat model of liver fibrosis

**DOI:** 10.1111/jcmm.13396

**Published:** 2017-11-03

**Authors:** Anna Cargnoni, Serafina Farigu, Ester Cotti Piccinelli, Patrizia Bonassi Signoroni, Pietro Romele, Graziella Vanosi, Ivan Toschi, Valentina Cesari, Luciana Barros Sant'Anna, Marta Magatti, Antonietta R. Silini, Ornella Parolini

**Affiliations:** ^1^ Centro di Ricerca E. Menni Fondazione Poliambulanza Istituto Ospedaliero Brescia Italy; ^2^ Dip. Scienze veterinarie per la salute, la produzione animale e la sicurezza alimentare Università di Milano Milano Italy; ^3^ Dip. Scienze Agrarie e Ambientali Università di Milano Milano Italy; ^4^ Institute of Research and Development University of Vale do Paraíba (UNIVAP) São José dos Campos São Paulo Brazil; ^5^ Istituto di Anatomia Umana e Biologia Cellulare Università Cattolica del Sacro Cuore Roma Italy

**Keywords:** placenta‐derived cells, human amniotic membrane, human amniotic cells, human amniotic epithelial cells, biliary liver fibrosis, bile duct ligation, myofibroblasts, ductular epithelial cells

## Abstract

Myofibroblasts are key fibrogenic cells responsible for excessive extracellular matrix synthesis characterizing the fibrotic lesion. In liver fibrosis, myofibroblasts derive either from activation of hepatic stellate cells (HSC) and portal fibroblasts (PF), or from the activation of fibroblasts that originate from ductular epithelial cells undergoing epithelial–mesenchymal transition. Ductular cells can also indirectly promote myofibroblast generation by activating TGF‐β, the main fibrogenic growth factor, through αvβ6 integrin. In addition, after liver injury, liver sinusoidal cells can lose their ability to maintain HSC quiescence, thus favouring HSC differentiation towards myofibroblasts. The amniotic membrane and epithelial cells (hAEC) derived thereof have been shown to decrease hepatic myofibroblast levels in rodents with liver fibrosis. In this study, in a rat model of liver fibrosis, we investigated the effects of hAEC on resident hepatic cells contributing to myofibroblast generation. Our data show that hAEC reduce myofibroblast numbers with a consequent reduction in fibronectin and collagen deposition. Interestingly, we show that hAEC strongly act on specific myofibroblast precursors. Specifically, hAEC reduce the activation of PF rather than HSC. In addition, hAEC target reactive ductular cells by inhibiting their proliferation and αvβ6 integrin expression, with a consequent decrease in TGF‐β activation. Moreover, hAEC counteract the transition of ductular cells towards fibroblasts, while it does not affect injury‐induced and fibrosis‐promoting sinusoidal alterations. In conclusion, among the emerging therapeutic applications of hAEC in liver diseases, their specific action on PF and ductular cells strongly suggests their application in liver injuries involving the expansion and activation of the portal compartment.

## Introduction

**Figure 1:**
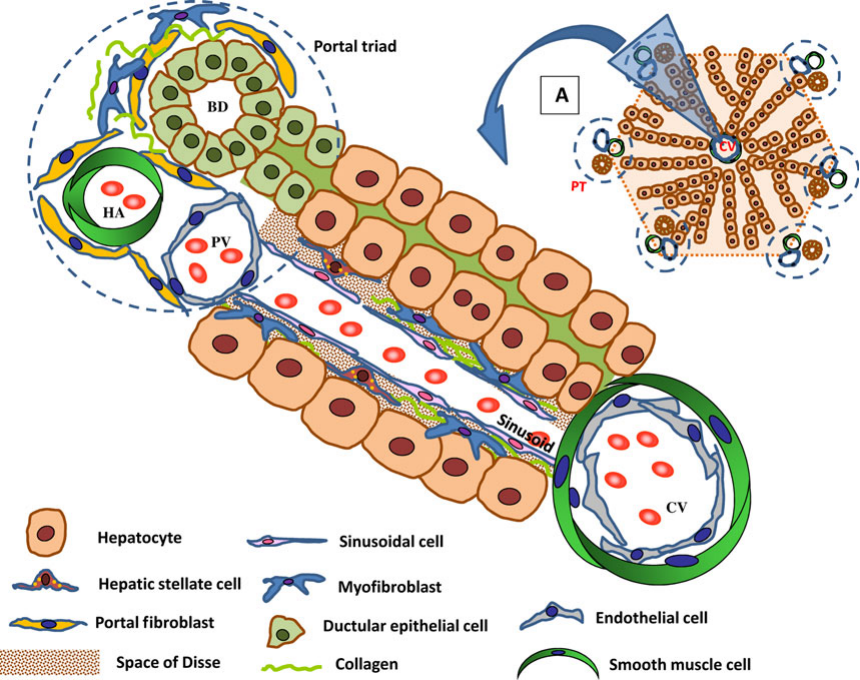
Liver structure and hepatic fibrogenic cells. The liver consists of lobules, representing the hepatic functional unit (panel **A**). Each lobule is intersected by a central vein (CV), from which hepatocyte cords radiate towards portal triads (PT) containing three different structures: bile ducts (BD), hepatic artery (HA) and portal vein (PV). Hepatocyte cords are separated by sinusoids, blood vessels lined by specialized fenestrated endothelial cells (liver sinusoidal cells). Hepatic stellate cells (HSC) are located in the space of Disse, and portal fibroblasts (PF) in the portal triad areas. Under injury conditions, both HSC and PF can be activated to myofibroblasts (ECM‐producing cells). Ductular epithelial cells, another cell type involved during liver injury, line the bile ducts.

Liver fibrosis, consequent to chronic liver injuries such as viral infections, alcohol and drug intoxications, metabolic disorders and cholestasis, is a major cause of morbidity and mortality worldwide 1. Liver fibrogenesis is a complex and dynamic process involving a heterogeneous population of fibrogenic hepatic cells. Among these, myofibroblasts are the key cells responsible for the excessive extracellular matrix (ECM) synthesis characterizing the fibrotic lesion, and their increased number correlates with the severity of liver fibrosis 2. Myofibroblasts derive from the activation of resident mesenchymal populations: HSC and PF (Fig. 1), or from epithelial (ductular epithelial cells) or endothelial (liver sinusoidal endothelial cells: LSEC) resident cells that directly or indirectly contribute to myofibroblast generation (Fig. 1).

Investigations mostly performed in rat 3, 4, 5 and mouse models 6, 7 of hepatic fibrosis demonstrated that HSC and PF activation towards myofibroblasts is consequent to a complex network of autocrine/paracrine fibrogenic signals, generated from an injury event, which promote up‐regulation of ECM proteins (*e.g*. fibronectin and collagen 1A1) and α‐smooth muscle actin (α‐SMA), conferring myofibroblasts with the ability to migrate to the site of injury 6, 8.

In the animal model of common bile duct ligation (BDL) applied in this study, which mimics obstruction to bile flow, such as that found in primary biliary cirrhosis and primary sclerosing cholangitis, PF play an important role in early ECM deposition around portal tracts 3, 4, 5, 6 and trigger HSC activation and migration (from the space of Disse to the injured area); HSC are instead crucial for the subsequent progression of the fibrotic lesion 6.

Furthermore, growing experimental evidence has shown that ductular epithelial cells (Fig. 1) also contribute to biliary fibrosis 9. In the early stage of biliary liver injury, ductular epithelial cells give rise to a composite reaction named ‘ductular reaction’ characterized by a marked expansion of the ductular compartment which adopts an inflammatory phenotype. Reactive ductular epithelial cells can undergo epithelial‐to‐mesenchymal transition (EMT), a dynamic process in which ductular cells lose their epithelial phenotype and gradually acquire a fibroblast and motile phenotype, with the potential to migrate out of the epithelial lining into the adjacent periportal stroma and transdifferentiate to myofibroblasts 9, 10. Reactive ductular epithelial cells can also indirectly promote myofibroblast generation by secreting several pro‐inflammatory and chemotactic cytokines (such as IL‐6 and MCP‐1) and growth factors (VEGF, PDGF, TGF‐β2, CTGF), which mediate the recruitment and activation of inflammatory and mesenchymal (HSC and PF) cells 11. Importantly, reactive ductular cells can activate TGF‐β, the key fibrogenic growth factor which drives fibroblast activation and ECM synthesis, through the expression of αvβ6 integrin on their own cell membrane 12.

Finally, LSEC (Fig. 1) represent another liver cell population with a role in the fibrogenic process 13. These cells are specific endothelial cells which, containing fenestrae and lacking the basement membrane, allow an efficient exchange of metabolites and oxygen between the blood and hepatocytes. Under normal conditions, they produce paracrine factors crucial to maintain HSC inside the space of Disse and under quiescent status 14. Under cholestatic injury, LSEC morphology and functionality are altered, they lose their characteristic fenestrae (process named ‘defenestration’) and importantly their ability in preserving HSC quiescence 14.

The study of the main fibrogenic cells contributing to liver injury and the mechanism(s) of activation in different fibrotic injury etiologies could provide new important targets to develop an effective antifibrotic therapy. The therapeutic options now under investigation include molecules that suppress/reduce reactive oxygen species (such as vitamin E), inflammatory mediators (anti‐TNF‐α, anti‐IL‐6 compounds) or that block angiotensin II (such as losartan), all of which are involved in HSC activation 15.

Among the proposed antifibrotic approaches, cell‐based therapies have been shown to reduce liver fibrosis in different animal models. We and others have demonstrated that the amniotic membrane and cells derived thereof, namely human amniotic mesenchymal stromal cells (hAMSC) and amniotic epithelial cells (hAEC), decrease hepatic fibrosis induced by BDL 16, 17, 18 and by CCl_4_ intoxication 19, 20, 21, 22.

Until now studies have been focused on the ability of amniotic cells either to favour hepatocyte proliferation/regeneration 20, 23 or to counteract fibrosis progression by modulating the inflammatory environment through the reduction in inflammatory cytokines 19 and the recruitment 21, activation 22 and differentiation 21 of hepatic macrophages.

However, studies aimed to examine the activity of hAEC on the resident hepatic cells that contribute to myofibroblast generation are lacking. Therefore, the aim of this study was to investigate the effects of hAEC on HSC, PF, epithelial ductular cells and LSEC in order to identify the specific target of hAEC treatment.

## Materials and methods

### Ethics statements

Human term placentas were collected after obtaining written informed consent according to the guidelines of the Ethical Committee of the Province of Brescia (*Comitato Etico Provinciale*).

Animal experiments were carried out in accordance with the guidelines established by the Italian law DL 26/2014 on the accommodation and care of animals used for scientific purposes. The experimental protocol was approved by Italian Ministry of Health (authorization n. 427/2015 PR) and by the Committee on the Ethics of Animal Experiments of the University of Milano (n. 105/14, 9/12/2014).

All animals received humane care according to the criteria outlined in the ‘Guide for the Care and Use of Laboratory Animals’ prepared by the National Academy of Sciences and published by the National Institutes of Health (NIH publication 86‐23 revised 1985).

### hAEC isolation

hAEC were isolated from term placentas (*n* = 5) as previously described 24. Briefly, the amnion was detached from chorion and washed in PBS (Sigma‐Aldrich, St Louis, MO, USA) supplemented with 100 U/ml penicillin (Lonza, Basel, Switzerland) and 100 μg/ml streptomycin (Sigma‐Aldrich). Amnion fragments ( ~15 × 15 cm) were incubated for 10 min. at 37°C in PBS containing 0.5 mM EDTA and P/S, and then in 1X trypsin/EDTA solution (Sigma‐Aldrich) for 5 min. at 37°C. After discarding debris, the fragments were again incubated (10 min. at 37°C) in fresh trypsin/EDTA solution and, after washing in PBS, were once more digested in trypsin/EDTA. The cells from the second and third digestions were pooled and centrifuged at 300× *g* for 10 min. Cell suspensions were then filtered through a 70‐μm cell strainer (BD Biosciences, San Jose, CA, USA), centrifuged and counted. Isolated cells were cryopreserved in 10% DMSO (Sigma‐Aldrich) supplemented with 90% FBS until use.

hAEC batches with cells over 95% positive for CD324 and CD166, and negative for CD44, CD105 and CD45 were used. Cell viability after thawing was always higher than 85%.

### Biliary fibrosis induction and animal treatment

All experimental procedures were performed on anaesthetized animals. Biliary fibrosis was induced by BDL in female Wistar rats weighing 220–250 g (Charles River Laboratories, Calco, Italy), as previously described 16, 18. Briefly, anaesthesia was induced with 5% isoflurane (IsoFlo^®^ Abbott Laboratories, Maidenhead, UK) and maintained at 2.5% during the surgical procedure. Before BDL, 4 mg/Kg Carprofen (Rimadyl^®^, Pfizer, Milano, Italy) and 5 mg/Kg Enrofloxacin (Baytril^®^, Bayer, Milano, Italy) were administered by subcutaneous injection to decrease pain and prevent infection. The common bile duct was exposed through a whole thickness laparoscopic midline incision, then ligated and sectioned to produce a permanent biliary obstruction. After BDL, 5 mg/kg vitamin K (Fitomenadione, Konakion^®^, Roche Products Limited, Welwyn Garden City, UK) was administered weekly to reduce haemorrhagic diathesis.

Immediately after BDL, animals were randomly treated through intratail vein (iv) injections, either with 400 μl PBS (BDL + PBS group) or with 3 × 10^6^ hAEC in 400 μl PBS (BDL + hAEC group). All animals were killed 6 weeks post‐surgery with an excess of isoflurane followed by puncture of the right ventricle and exsanguination.

### Serum bilirubin

Total bilirubin concentrations were determined in serum collected from animals 7 days after BDL, by the diazo method using Bilirubina totale, metodo colorimetrico (DMSO) Kit (Giesse Diagnostic snc, Roma, Italy) according to the manufacturer's recommendations.

### Liver histological examination

At sacrifice, a portion of the hepatic median lobe from each animal was immediately fixed in 4% formalin for 48 hrs and paraffin‐embedded.

To assess the degree of liver fibrosis, 4‐μm‐thick sections were stained with haematoxylin/eosin and Goldner's modified Masson trichrome, according to the manufacturer's instructions (BiOptica, Milano, Italy). Histological grading of liver fibrosis was evaluated on microscopic fields centred on hepatic lobules, under a bright field microscope (Olympus BX41, Tokyo, Japan) at 100× magnification, by the semiquantitative Knodell's scoring system 25 performed by a pathologist kept blinded of the treatment applied. This score system allows a staging of liver fibrotic lesions considering the progressive expansion of the collagen deposits, from portal regions to gradually involving the interstitial parenchyma and finally resulting in liver remodelling 16, 17. The average of the score taken from 10 to 12 non‐overlapping random fields per section was used to generate a single score for each animal.

To evaluate the extent of liver collagen deposition, sections were incubated in PicroSirius Red (PSR; Sigma‐Aldrich; Direct Red 80, 0.1% wt/vol in saturated picric acid) for 80 min. and washed for 3 min. in tap water. Twelve non‐overlapping fields were acquired (200× magnification), images digitized and PSR‐positive area measured by computer‐assisted morphometric analysis using FiJi software (https://imagej.nih.gov/ij).

### Immunohistochemistry

Serial liver sections of each specimen were immuno‐stained for α‐SMA (1:600; Clone 1A4, Dako, Glostrup, Denmark); fibronectin (1:100, clone 10/Fibronectin, BD Transduction Laboratories, Franklin Lakes, NJ, USA); desmin (1:1,000, Gene‐Tex, Aachen, Germany); collagen 15A1 (1:100, clone N20, Santa Cruz Biotechnology, Dallas, Tx, USA); cytokeratin 19 (CK19) (1:100, clone‐b170, Leica Biosystems, Newcastle, UK); αvβ6 integrin (1:150, Bioss Inc. Woburn, MA, USA); S100A4, also called fibroblast‐specific protein‐1 (FSP‐1) (1:300, Dako); phosphorylated Smad 2/3 (p‐Smad2/3) (1:200, Santa Cruz Biotechnology); CD32b (1:500, Sino Biological, Beijing, China) and CD34 (1:50, Bioss Inc.).

Briefly, dewaxing and antigen retrieval was performed at 98°C for 20–30 min. in a citrate buffer solution pH 6 (BiOptica); a further digestion of 10 min. in trypsin solution (Invitrogen Corporation, Frederick, MD, USA) at 37°C was necessary for CK19 detection.

Endogenous peroxidase activity was quenched by 10‐min. incubation with Dako REAL Peroxidase‐Blocking Solution (Dako), and unspecific labelling was minimized by a 30‐min. incubation with 2.5% normal horse serum (ImmPRESS reagent kit, Vector Laboratories, Burlingame, CA, USA) or with 10% normal goat serum (Invitrogen Corporation).

Sections, incubated overnight at 4°C with one of the primary antibodies, were then washed and incubated with an horseradish peroxidase (HRP)‐conjugated secondary antibody (ImmPRESS reagent kit, rat absorbed, Vector Laboratories) or with a biotinylated secondary antibody (Vector Laboratories) (fibronectin, collagen 15A1, p‐Smad2/3 and CD32b). In this last case, antibody binding was detected by Streptavidin‐HRP (Vector Laboratories). Diaminobenzidine (ImmPACT DAB, Vector Laboratories) was used as the chromogen.

Slides were counterstained with haematoxylin and mounted. Substitution of primary antibody with an irrelevant IgG served as a negative control.

For each marker, 10–18 single images at 200× magnification were taken from each tissue section with a digital camera (Olympus Camedia C‐4040 ZOOM), yielding homogeneous 24 bit colour images with a 1024 × 768 pixel size and 200 dpi resolution. The % of area positive for each marker was calculated by computer‐assisted morphometric analysis using FiJi software (https://imagej.nih.gov/ij).

### Polymerase chain reaction

Total RNA was extracted from snap‐frozen portions of the hepatic median lobe using EZ1 RNA Universal Tissue Kit (Qiagen, Hilden, Germany) following manufacturer's instructions. One hundred nanograms (2 μl) of total RNA was retrotranscribed to cDNA using the ImProm‐II™ ReverseTranscription System (Promega, Madison, WI, USA) and amplified by polymerase chain reaction (PCR) in a final volume of 50 μl, using Go Taq Polymerase (Promega). The nucleotide sequences of primers used in the PCR are listed in Table 1. Samples were denatured at 95°C for 10 min., then exposed to 25 (collagen1A1)/28 (CK19)/30 (desmin; fibronectin; α‐SMA; S100A4) or 32 (collagen15A1; αvβ6 integrin) cycles of denaturation (at 95°C for 30 sec.), annealing at 54°C (fibronectin; αvβ6 integrin)/56°C (collagen 15A1; α‐SMA; CK19; S100A4) or 58°C (collagen 1A1; desmin) for 30 sec., and elongation (72°C for 60 sec.) followed by a final elongation at 72°C for 7 min.

**Table 1:** Primer sequences for RT‐PCR

Genes	Primer sequence (5′‐3′)	Tm (°C)	Size (bp)
α‐SMA	F: TGCTCCAGCTATGTGTGAAGA	56	325
	R: AGGTCGGATGCTCCTCTG		
Fibronectin	F: CCAGGCACTGACTACAAGAT	54	145
	R: CATGATACCAGCAAGGACTT		
Desmin	F: GACCTAGAGCGCAGAATTGAGT	58	476
	R: GCCATCTCATCCTTTAGGTGTC		
Collagen 15A1	F: GCCCCCTACTTCATCCTCTC	56	154
	R: CAGTACGGACCTCCAGGGTA		
Collagen 1A1	F: TGAGCCAGCAGATTGAGAACA	58	120
	R: GGGTCGATCCAGTACTCTCCG		
S100A4	F: ATACTCAGGCAACGAGGGTG	56	245
	R: CTTCCGGGGCTCCTTATC		
CK19	F: ACACCAGGCATTGACCTAGC	56	279
	R: TCCGTAACGGGCCTCTATCT		
αvβ6 Integrin	F: ATCCTGCTCATCGGTGTCG	54	188
	R: CTTTGTGCTTCTCCCTGTTTG		
Housekeeping genes			
β‐actin	F: GAGAGGGAAATCGTGCGTGAC		452
	R: CCCATACCCACCATCACACC		
18S rRNA	F: GAGCGAAAGCATTTGCCAAG		100
	R: GGCATCGTTTATGGTCGGAA		

Housekeeping genes (β‐actin or 18S rRNA) were used as internal controls to calculate relative quantification of target gene expression. Target and housekeeping genes were amplified in the same reaction.

The amplified PCR products were stained with ethidium bromide on a 1.5% agarose gel and visualized by UV light and photographed using UV transilluminator GelDoc 2000 system (Bio‐Rad Laboratories S.r.l., Segrate, Italy). Gels were analysed by ImageJ software (http://rsb.info.nih.gov/ij/docs/menus/analyze.html#gels) to calculate the intensity values of strips.

### Statistical analysis

Data are expressed as median values and the relative interquartile range (IQR). Differences between experimental groups (PBS and hAEC groups) were assessed by nonparametric Mann–Whitney test. A *P* value < 0.05 was considered statistically significant. Statistical analysis was performed with GraphPad Prism 6 Software (GraphPad Software, San Diego, CA, USA).

## Results

### hAEC treatment reduces extracellular matrix deposition

Liver fibrosis can be defined as a result of the progressive accumulation and remodelling of the ECM, which disrupts the normal hepatic architecture.

In this study, liver fibrosis was induced in rats by BDL. The presence of jaundice (2 days post‐ligation) and high serum concentrations of total bilirubin (ranged between 118.3 and 273.1 μmol/l in BDL‐injured animals versus 0.3 and 1.2 μmol/l in non‐injured ones) indicated a successful obstruction.

ECM presence in healthy livers was very low. Fibronectin was observed within the portal area and around the central vein (the percentage of area occupied by fibronectin‐positive cells in sections from healthy livers was between 0.5 and 1.3%); collagen was present in very low amounts in the periportal area as a thin rim around blood vessels.

**Figure 2:**
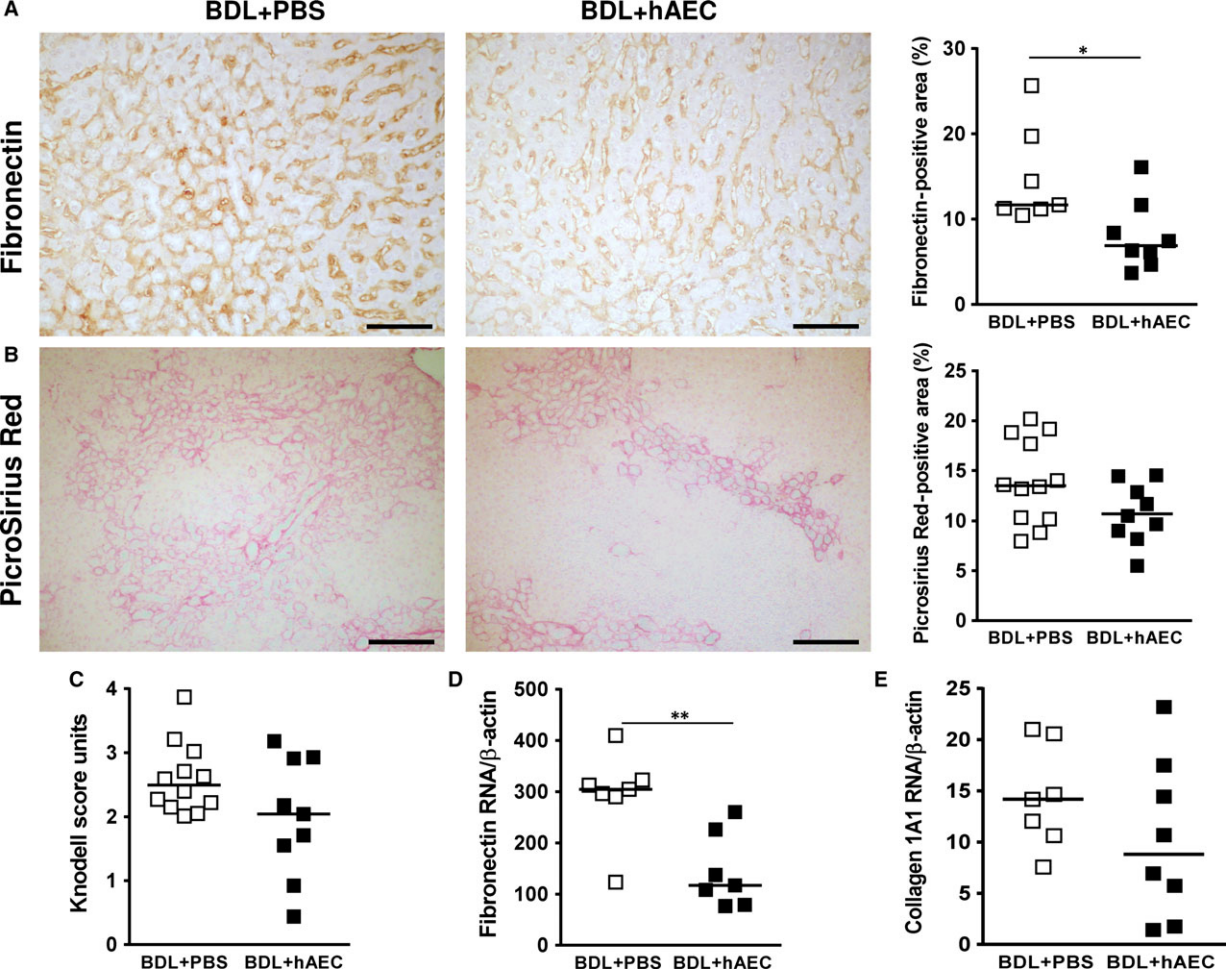
hAEC treatment reduces extracellular matrix deposition. Livers extracted from control (BDL + PBS) and treated (BDL + hAEC) rats, 6 weeks after bile duct ligation (BDL), were evaluated for extracellular matrix protein (fibronectin and collagen) deposition and the degree of fibrosis. Representative microphotographs of fibronectin immuno‐staining and collagen staining (PicroSirius Red: PSR) are reported in panels (**A**) and (**B**). hAEC‐treated rats showed lower hepatic areas, quantified by computer‐assisted image analysis, positive for fibronectin (**A**) and PSR (**B**), and reduced liver fibrosis in terms of Knodell's score units (**C**). Similar results were obtained for fibronectin (**D**) and collagen 1A1 (**E**) mRNA liver expression. Scale bars = 100 μm (**A**), 200 μm (**B**). *P < 0.05 and **P < 0.01, *versus* BDL + PBS group.

On the other hand, livers extracted from control animals (BDL + PBS group) 6 weeks after BDL showed a large amount of ECM. Fibronectin was arranged around biliary ducts and along hepatic sinusoids (Fig. 2A). Collagen deposits were mainly found around the biliary ductular structures forming bridging septa which link adjacent portal areas and infiltrated liver parenchyma connecting portal areas and the lobule central vein (Fig. 2B).

To evaluate the effects of hAEC treatment, the degree of fibrosis in livers from control and treated animals was determined by analysing fibronectin deposition, collagen accumulation (through quantitation of collagen‐positive area on PSR‐stained liver sections) and collagen distribution in hepatic lobules (through Knodell's score system applied to Masson's stained liver sections, see Material and Methods section).

We observed that hAEC treatment significantly decreased fibronectin deposition [from 11.67 (IQR: 8.53) to 6.88 (IQR: 5.82) % liver‐positive area, *P* < 0.05] (Fig. 2A). It also reduced both collagen accumulation [10.50 (IQR: 5.06) *versus* 13.47 (IQR: 8.32), % liver area occupied by PSR‐positive staining] (Fig. 2B) and collagen distribution [2.04 (IQR: 1.69) *versus* 2.50 (IQR: 0.78) score units in treated and control rats, respectively] (Fig. 2C). These results were in line with fibronectin and collagen 1A1 mRNA liver expression (Fig. 2D and E).

### hAEC decrease hepatic fibroblast (PF and HSC) activation

**Figure 3:**
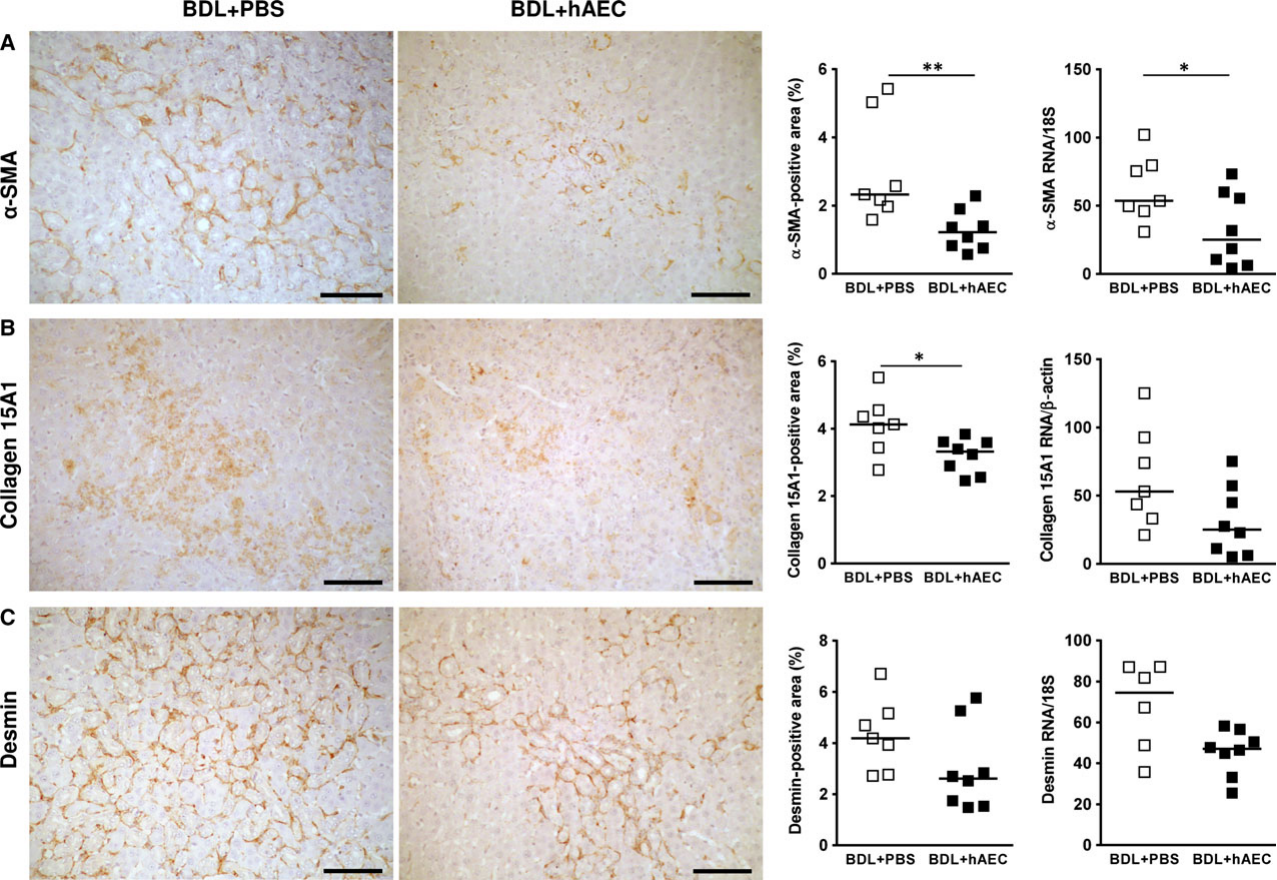
hAEC treatment decreases hepatic fibroblast activation. Immunohistochemical and quantitative image analysis of rat liver sections from control (BDL + PBS) and treated (BDL + hAEC) rats, 6 weeks after BDL. In hAEC‐treated rats, the percentages of liver areas occupied by activated myofibroblasts (α‐SMA‐positive cells) (**A**), by myofibroblasts derived from portal fibroblasts (PF) (collagen 15A1‐positive liver areas) (**B**) and by myofibroblasts derived from hepatic stellate cells (HSC) (desmin‐positive liver areas) (**C**), are reduced compared to the control (BDL + PBS) group. Similar results were obtained from the analysis of mRNA expression (panels **A**,** B** and **C**). Scale bars = 100 μm. *P < 0.05 and **P < 0.01 *versus* BDL + PBS group.

Myofibroblasts are characterized by a contractile phenotype and typically express α‐SMA. In liver sections from healthy rats, α‐SMA expression is very low and limited to the wall of portal vessels. As expected, in livers from fibrotic rats, α‐SMA‐positive cells are instead located also around biliary ducts, thus in the same areas occupied by fibronectin and collagen deposits. hAEC‐treated animals showed a significant lower area occupied by myofibroblasts with respect to livers from control animals [1.22 (IQR: 1.01) *versus* 2.33 (IQR: 3.06) % liver‐positive area for α‐SMA, *P* < 0.01] (Fig. 3A).

As myofibroblasts can derive from the activation of PF and HSC, we examined whether hAEC can impact the activation of these cells. We therefore investigated the phenotype of myofibroblasts arising from PF activation as collagen 15A1‐positive cells 4, and those arising from HSC activation as desmin‐positive cells 6. We also analysed collagen 15A1 and desmin mRNA expression in whole liver extracts.

In liver sections of healthy rats, very rare collagen 15A1‐positive cells were present in the periportal areas. Similarly, desmin staining was very scant, and rare positive cells (0.5–0.9% of the total section area) were restricted to arterial walls and along sinusoids.

The presence of these markers increased in BDL‐injured rats.

It is noteworthy that hAEC displayed a significant inhibition on myofibroblasts arising from PF activation [3.32 (IQR: 0.96) *versus* 4.13 (IQR: 1.11) % collagen 15A1‐positive area, *P* < 0.05] (Fig. 3B). hAEC‐treated animals also showed a reduced number of myofibroblasts deriving from HSC, although this effect was not significant [2.61 (IQR: 3.07) *versus* 4.18 (IQR: 2.39) % desmin‐positive area in hAEC‐treated and control groups, respectively; *P* = 0.1203] (Fig. 3C).

Data collected from PCR analysis were in line with those obtained from IHC analysis. (Fig. 3A–C).

### hAEC reduce the fibrogenic activity of hepatic ductular epithelial cells

We then addressed the hypothesis that hAEC may reduce myofibroblast generation by acting on ductular epithelial cells. In healthy livers, ductular structures were limited and present only in the portal areas (in our staining condition, they occupied about 0.6–1.0% of the total section area). In response to biliary obstruction, ductular epithelial cells intensively proliferate and give rise to increased numbers of biliary structures (‘ductular reaction’).

**Figure 4:**
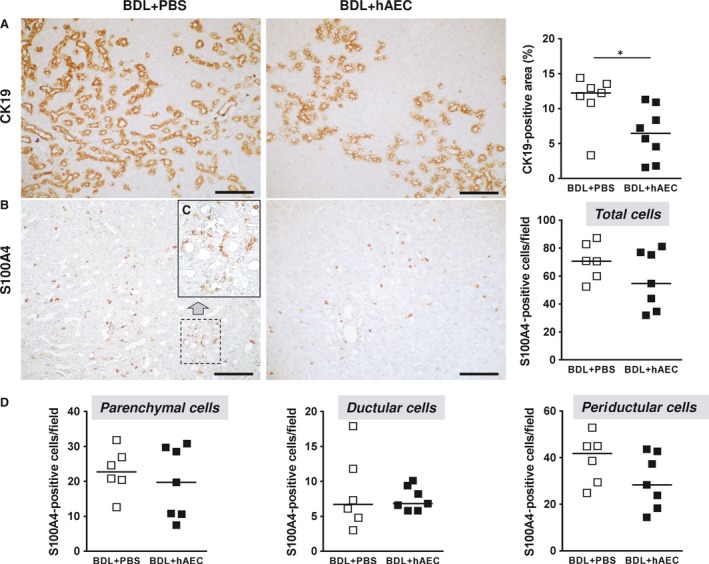
hAEC treatment reduces the ductular reaction and EMT. Immunohistochemical and quantitative image analysis of rat liver sections from control (BDL + PBS) and treated (BDL + hAEC) rats, 6 weeks after BDL. Livers from hAEC‐treated rats exhibited a significant decrease in liver area occupied by ductular epithelial cells (positive for CK19) (**A**) and a lower number of cells undergoing EMT (positive for S100A4) (**B**). The treatment did not affect the number of positive cells located in parenchymal and ductular areas, but rather those close to ductular structures (periductular cells) (**C** and **D**). Scale bars = 100 μm (**A** and **B**) and 50 μm (**C**). *P < 0.05 *versus* BDL + PBS group.

We first assessed whether hAEC treatment could limit the ductular reaction by detecting CK19‐positive cells, a marker which is normally expressed by ductular epithelial cells (Fig. 4A). hAEC‐treated animals showed a severe and significant decrease in CK19 protein expression [12.24 (IQR: 2.71) *versus* 6.47 (IQR: 7.79), *P* < 0.05] (Fig. 4A) and mRNA expression [100.8 (IQR: 49.9) *versus* 38.5 (IQR: 39.8), *P* < 0.05] with respect to control animals.

We further investigated whether hAEC could inhibit the possible transition of ductular epithelial cells towards a fibroblast phenotype. For this purpose, we assessed hepatic cells expressing S100A4 (also called FSP‐1), a marker of epithelial–mesenchymal transition 10, 26. In livers from fibrotic control animals, the number of S100A4‐positive cells ranged from 52.4 to 87.2 in each analysed field, most of these cells (more than 50%) were located in the periductular areas (Fig. 4B–D). Livers from hAEC‐treated animals exhibited a lower number of cells positive for S100A4 [54.6 (IQR: 42.2) *versus* 70.7 (IQR: 25.9)] (Fig. 4B). The treatment did not affect the number of positive cells located in ductular and parenchymal areas, but rather those close to ductular structures (periductular cells) (Fig. 4D). In hAEC‐treated animals, there were 28.3 (IQR: 24.4) periductular S100A4‐positive cells/field *versus* 41.8 (IQR: 18.7) counted in control livers (*P* = 0.1014).

**Figure 5:**
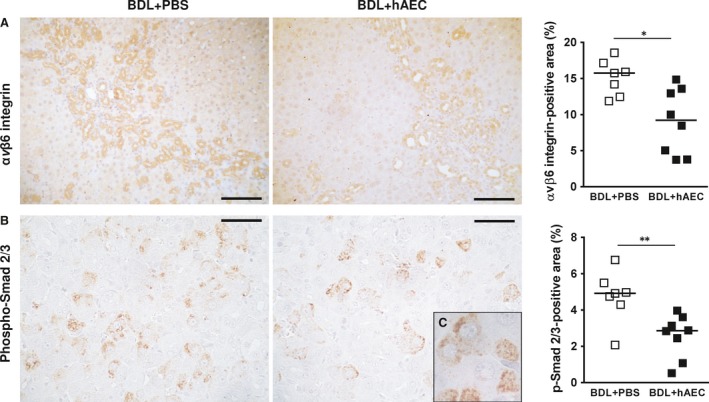
hAEC treatment decreases TGF‐β pathway activation. Immunohistochemical and quantitative image analysis of rat liver sections from control (BDL + PBS) and treated (BDL + hAEC) rats, 6 weeks after BDL. Representative microphotographs related to: (**A**) αvβ6 integrin ductular expression—up‐regulated under BDL conditions—and (**B**) phosphorylated Smad2/3 expression—identifying cells with activated TGF‐β pathway. hAEC‐treated rats show a lower hepatic area occupied by αvβ6 integrin (**A**) associated with a strong reduction in p‐Smad2/3‐positive area (**B**). p‐Smad2/3 signals are located in perinuclear and nuclear areas indicating both ongoing and completed translocations of p‐Smad2/3 to the nucleus (*in box* **C**, 1000× magnification). Scale bars = 100 μm (**A**) and 50 μm (**B**). *P < 0.05 and **P < 0.01 *versus* BDL + PBS group.

Reactive ductular cells can also indirectly promote fibroblast differentiation towards myofibroblasts by activating latent TGF‐β through up‐regulation of αvβ6 integrin expressed on their cell membrane 11, 12; of note in sections from healthy livers, ductular cells did not express αvβ6 integrin. We explored whether hAEC treatment reduced TGF‐β pathway activation *via* inhibition of ductular expression of αvβ6 integrin. We first observed that αvβ6 integrin expression was lower in livers of hAEC‐treated animals [9.22 (IQR: 9.32) *versus* 15.75 (IQR: 4.68) % liver‐positive area for αvβ6 integrin; *P* < 0.05] (Fig. 5A). We then verified whether this effect was associated with TGF‐β pathway inhibition. To this end, we used phosphorylated Smad 2/3 (p‐Smad2/3) expression as an indicator of TGF‐β activation as p‐Smad2/3 proteins act as pivotal downstream effectors of TGF‐β by transferring signals from TGF‐β receptors to the nucleus. In hAEC‐treated animals, in concert with the reduction in αvβ6 integrin expression, we observed a strong and significant reduction in p‐Smad2/3‐positive cells [2.86 (IQR: 2.07) *versus* 4.92 (IQR: 1.20) % liver‐positive area for p‐Smad2/3; *P* < 0.01] (Fig. 5B).

### hAEC do not act on liver sinusoidal endothelial cell defenestration

**Figure 6:**
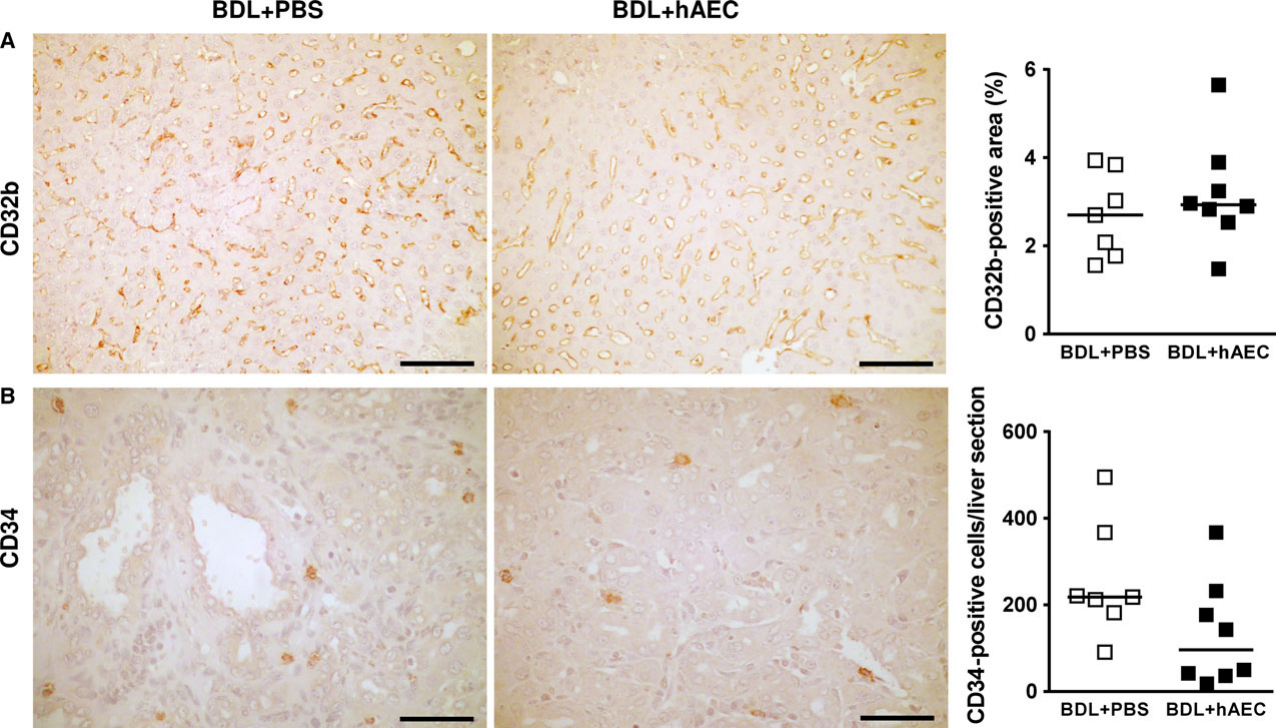
hAEC treatment does not act on liver sinusoidal endothelial cell defenestration. Immunohistochemical and quantitative image analysis of rat liver sections from control (BDL + PBS) and treated (BDL + hAEC) rats, 6 weeks after BDL. Representative microphotographs of: (**A**) CD32b expression in sinusoidal cells—the expression of this protein correlates with the presence of fenestrations in these cells—and (**B**) CD34 expressing cells—shown to be possibly increased during sinusoidal defenestration/capillarization. hAEC treatment does not affect BDL‐induced sinusoidal defenestration, but modestly decreases the number of CD34‐expressing cells. Scale bars = 100 μm (**A**) and 50 μm (**B**).

Finally, we investigated the ability of hAEC to reduce sinusoidal ‘capillarization’, a process thought to be involved in liver fibrosis development and HSC activation 13, 14. This process results in alteration of LSEC phenotype with loss of their ‘fenestrae’ (defenestration) and basement membrane formation. To evaluate the effect of hAEC on LSEC, we analysed the expression of CD32b, a cell surface protein that correlates with the presence of cell fenestrations 27. As expected, BDL induced a reduction in LSEC fenestrations; in fact, livers from healthy animals showed an area positive for CD32b ranging between 7.1 and 13.8% of the total section area, while it was reduced to 1.6 and 3.9% in BDL animals. Our data show that hAEC did not affect BDL‐induced sinusoidal defenestration; CD32b expression was similar in animals from both BDL + PBS and BDL + hAEC groups [2.70 (IQR: 2.07) *versus* 2.93 (IQR: 1.12) % liver‐positive area for CD32b, respectively; *P* = 0.593] (Fig. 6A).

Although hAEC treatment does not affect LSEC defenestration, it slows the number of CD34‐expressing cells (Fig. 6B), which have been shown to increase during capillarization 28.

## Discussion

We and others have previously demonstrated that amniotic membrane and hAEC decrease liver fibrosis in terms of reduction in myofibroblast levels and collagen deposition 16, 18, 19, 21. Considering the fundamental role of myofibroblasts in the development and progression of the fibrotic lesion, in this study, we aimed to narrow down the actions of hAEC on different myofibroblast precursors, namely HSC, PF, epithelial ductular cells and LSEC.

Myofibroblasts, the key fibrogenic cells responsible for ECM synthesis and deposition, derive from the activation of PF and HSC, two liver fibroblast populations with distinctive roles in the biliary fibrotic process. PF are the first responders to liver injury caused by biliary obstruction, and their activation is an initial event responsible for early ECM deposition and for triggering HSC migration and activation 6. Activated HSC are instead responsible for the fibrosis progression.

In this study, not only do we show that hAEC significantly reduce liver myofibroblast levels, but also for the first time we show that hAEC exert a stronger inhibition on PF activation rather than on HSC activation, suggesting that cell treatment affects the cells involved in the early events of the fibrotic process and located in the portal area, which is directly affected by biliary occlusion. In line with the more robust actions of hAEC on PF, we observed a robust decrease in fibronectin levels which could be justified by previous studies which have shown that myofibroblasts derived from PF produce higher levels of fibronectin with respect to myofibroblasts derived from HSC 3, 4.

We furthermore investigated the ability of hAEC to limit the pro‐fibrogenic activity of epithelial ductular cells that also participate to myofibroblast generation.

Indeed, these cells may give rise to an early reaction (‘ductular reaction’) characterized by a strong ductular proliferation and acquisition of an inflammatory phenotype leading to the secretion of inflammatory mediators promoting the activation of periductular fibroblasts towards myofibroblasts. Our data demonstrate that hAEC strongly decrease ductular proliferation and thus possibly contribute in reducing the inflammatory milieu generated by the reactive ductular cells and consequently their pro‐fibrogenic activity. Under BDL‐induced inflammatory conditions, αvβ6 integrin is up‐regulated on the ductular surface 7, and it represents a key link between ductular inflammation and biliary fibrotic process. This integrin is indeed directly involved in liver fibrogenesis as it locally (in periductular areas) activates TGF‐β 12, 29, the main fibrogenic factor, which, under normal conditions is extracellularly stored in its inactive latent form unable to bind to its receptors. The crucial role of αvβ6 integrin in TGF‐β activation has been demonstrated in biliary fibrosis models where its neutralization has been shown to decrease fibrosis progression 12, 30, 31.

Importantly, we observed that hAEC treatment effectively decreases ductular expression of αvβ6 integrin, and this action is associated to a significant reduction in TGF‐β activation.

Interestingly, given the involvement of fibronectin in TGF‐β activation mediated by αvβ6 integrin 29, the lower fibronectin deposition observed in livers from hAEC‐treated animals could also contribute to maintaining TGF‐β in its inactivated, bound‐latent form.

Furthermore, as αvβ6 integrin has been shown to induce the proliferation of different cells 32, 33, the inhibition of ductular αvβ6 expression could also contribute to the reduced proliferation of reactive ductular cells.

In addition to ‘ductular reaction’, after liver injury, ductular epithelial cells may undergo epithelial‐to‐mesenchymal transition giving rising to EMT‐derived fibroblasts which potentially differentiate towards ECM‐producing myofibroblasts. We found that hAEC treatment reduces the number of EMT‐derived periductular fibroblasts (S100A4‐positive cells) 10, 26, but it remains to be clarified whether this reduction could contribute to the decreased myofibroblast levels observed after hAEC treatment 34.

Finally, on the basis of the ability of the amniotic membrane to maintain liver sinusoidal cell fenestrations during *in vitro* culture 35, we explored whether hAEC could retain this ability in BDL, a condition which also induces sinusoidal loss of fenestration and ‘capillarization’. Instead, we found that hAEC treatment does not limit sinusoidal cell defenestration, but only appears to slow CD34 expression which has been reported to increase during the ‘capillarization’ process 28. The finding that hAEC do not seem to target sinusoidal cells, although involved in the early fibrotic process, could be attributed to the fact that these are not highly proliferating cells and thus may be less susceptible to the antiproliferative action of hAEC 36, 37, 38, 39, 40.

In conclusion, our data show that hAEC reduce PF activation towards myofibroblasts and the ability of reactive ductular cells to generate periductular myofibroblasts. Thus, our results suggest that hAEC act on pro‐fibrogenic cells located in portal area and directly involved in biliary injury, which display an early, proliferative activity and an inflammatory phenotype. Finally, these data suggest the application of hAEC as a possible therapy not only in biliary‐associated fibrosis, but also in any liver injury which involves the expansion and activation of the ductular epithelial compartment.

## Author contributions

A.C. participated in the design of the study, performed the experiments, analysed the data and drafted the manuscript; S.E.F. and L.B.S. carried out microscopy analysis; E.C.P. and P.R. processed placental amniotic membranes to collect hAEC; P.B.S. and E.C.P. performed molecular analysis; G.V. performed animal surgery; I.T. and V.C. were responsible for animal care; M.M. and A.S. participated in the drafting of the manuscript; O.P. conceived of the study and participated in its design, supervised the research, participated in drafting and critically revised the manuscript. All authors read and approved the final version of the manuscript.

## Conflict of interest

The authors confirm that there is no conflict of interests.
